# Topical pH Sensing NIR Fluorophores for Intraoperative Imaging and Surgery of Disseminated Ovarian Cancer

**DOI:** 10.1002/advs.202201416

**Published:** 2022-05-14

**Authors:** Shinya Yokomizo, Maged Henary, Emmanuel R. Buabeng, Takeshi Fukuda, Hailey Monaco, Yoonji Baek, Sophia Manganiello, Haoran Wang, Jo Kubota, Amy Daniel Ulumben, Xiangmin Lv, Cheng Wang, Kazumasa Inoue, Masahiro Fukushi, Homan Kang, Kai Bao, Satoshi Kashiwagi, Hak Soo Choi

**Affiliations:** ^1^ Gordon Center for Medical Imaging Department of Radiology Massachusetts General Hospital and Harvard Medical School Boston MA 02114 USA; ^2^ Department of Radiological Sciences Tokyo Metropolitan University 7‐2‐10 Higashi‐Ogu Arakawa Tokyo 116–8551 Japan; ^3^ Department of Chemistry and Center for Diagnostics and Therapeutics Georgia State University 100 Piedmont Avenue SE Atlanta GA 30303 USA; ^4^ Department of Obstetrics and Gynecology Osaka City University Graduate School of Medicine 1‐4‐3, Asahimachi Abeno‐ku Osaka 545–8585 Japan; ^5^ Vincent Center for Reproductive Biology Vincent Department of Obstetrics and Gynecology Massachusetts General Hospital Boston MA 02114 USA

**Keywords:** fluorescence‐guided surgery, near‐infrared imaging, organic‐anion‐transporting polypeptides, structure inherent targeting, tumor microenvironment

## Abstract

Fluorescence‐guided surgery (FGS) aids surgeons with real‐time visualization of small cancer foci and borders, which improves surgical and prognostic efficacy of cancer. Despite the steady advances in imaging devices, there is a scarcity of fluorophores available to achieve optimal FGS. Here, 1) a pH‐sensitive near‐infrared fluorophore that exhibits rapid signal changes in acidic tumor microenvironments (TME) caused by the attenuation of intramolecular quenching, 2) the inherent targeting for cancer based on chemical structure (structure inherent targeting, SIT), and 3) mitochondrial and lysosomal retention are reported. After topical application of **PH08** on peritoneal tumor regions in ovarian cancer‐bearing mice, a rapid fluorescence increase (< 10 min), and extended preservation of signals (> 4 h post‐topical application) are observed, which together allow for the visualization of submillimeter tumors with a high tumor‐to‐background ratio (TBR > 5.0). In addition, **PH08** is preferentially transported to cancer cells via organic anion transporter peptides (OATPs) and colocalizes in the mitochondria and lysosomes due to the positive charges, enabling a long retention time during FGS. **PH08** not only has a significant impact on surgical and diagnostic applications but also provides an effective and scalable strategy to design therapeutic agents for a wide array of cancers.

## Graphical abstract

**PH08** shows intrinsic targetability to organic anion transporters, which are often overexpressed in cancer cells, and are taken up by tumor cells via endocytosis. The rapid reduction in intramolecular quenching occurs in the acidic tumor microenvironment and lysosomes of cancer cells, realizing enhanced fluorescence emission and a high tumor‐to‐background ratio to detect small peritoneal dissemination of ovarian cancer during intraoperative fluorescence imaging.

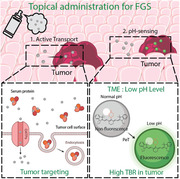

## 1 Introduction

Ovarian cancer is typically diagnosed at a late stage, which usually includes widespread peritoneal dissemination due to a lack of effective screening methods.^[^ ^1^ ^]^ Successful staging to map the localization of ovarian cancer and cytoreduction during surgery has been proven to significantly impact the prognosis of patients.^[^ ^2^, ^3^, ^4^, ^5^, ^6^ ^]^ One of the most significant prognostic factors is the amount of residual tumor after surgery.^[^ ^4^ ^]^ Therefore, it is crucial to reduce the tumor burden as much as possible, even when complete resection is impossible. However, accurate mapping and cytoreductive surgery for peritoneally‐disseminated ovarian cancer, with numerous submillimeter lesions in multiple anatomical sites on the peritoneal surface, is often challenging and could lead to poor treatment outcomes and recurrence.^[^ ^7^ ^]^

Fluorescence‐guided surgery (FGS) can be useful for ovarian cancer when a tumor‐targeted fluorophore is used to localize small tumor lesions with sufficient specificity and sensitivity, resulting in successful surgical resection and minimal operating room time, and obviating the need for repeated operations.^[^ ^8^, ^9^, ^10^, ^11^, ^12^, ^13^ ^]^ FGS has been approved for various procedures, including identifying tumor margin, sentinel lymph node mapping, angiography, and lymphography,^[^ ^14^ ^]^ and has improved tumor resection rates while minimizing normal tissue damage.^[^ ^15^, ^16^ ^]^ Particularly, near‐infrared (NIR) fluorescence imaging has led the field due to the reduced scattering, minimal tissue absorption, high tissue penetration depth, and low tissue autofluorescence interference, offering a high signal‐to‐background ratio (SBR) for deep tissue imaging.^[^ ^8^, ^9^, ^10^, ^11^ ^]^

In response to this, many tumor‐targeted NIR probes have been actively developed for FGS.^[^ ^12^, ^17^, ^18^, ^19^, ^20^, ^21^, ^22^, ^23^ ^]^ Particularly, “activatable” fluorophores hold promise in FGS by minimizing the background signal originating from non‐target tissues and achieving elevated fluorescence signals upon targeting.^[^ ^22^, ^24^, ^25^, ^26^, ^27^ ^]^ “Always on” fluorescent probes usually require a considerably long time to clear from the background tissues to attenuate high background signal for targeted imaging, whereas activatable fluorescent probes typically show lower background signals resulting in higher tumor‐to‐background ratios (TBRs) upon activation and can recognize cancer‐specific environments without washing out the unbound probes.^[^ ^8^, ^9^, ^10^, ^11^ ^]^ To this end, many rationally designed activatable fluorophores, especially those based on the concept of intramolecular photoinduced electron transfer (PeT), have been developed.^[^ ^28^, ^29^ ^]^ In particular, acidic tissue microenvironments and intracellular compartments have been targeted for activatable probes.^[^ ^30^, ^31^ ^]^ A hallmark of the tumor microenvironment (TME) is the acidic condition created by altered metabolism (a.k.a., the Warburg effect).^[^ ^32^, ^33^, ^34^ ^]^ Typically, non‐cancerous cells display an extracellular pH of around 7.5, while tumor cells show 6.4–7.1.^[^ ^35^ ^]^ Since imaging probes generally lack endogenous targeting mechanisms, they have often been combined with a targeting moiety to enhance contrast, such as antibodies which are generally large in molecular weight and show slow clearance. This strategy results in a long wait time between administration and imaging (up to days) before a sufficiently high SBR is obtained,^[^ ^36^ ^]^ making this approach less attractive for FGS. In contrast, small molecules with molecular weights 10–1000 times less than peptides and proteins can distribute rapidly to their targets with fast elimination of off‐target molecules, which renders high SBR.^[^ ^37^ ^]^ In addition, this approach is suitable for their clinical translation with economical, large‐scale, and reproducible production. Remarkably, Tung et al. overcame this limitation by establishing molecular imaging of intraperitoneal dissemination of ovarian cancer with topical application of a rationally designed activatable NIR fluorophore detecting mildly low pH based on PeT.^[^ ^19^ ^]^ This heptamethine fluorophore was small and could readily diffuse into tumor tissue. This approach has been reported to successfully visualize tiny tumors 3 h after the intraperitoneal injection in vivo, demonstrating the promise and feasibility of this approach. Towards its clinical translation, however, there is still room for improvements such as shorter staining times and extended tissue retention of the fluorescence signal for rapid and durable imaging for FGS.

In this study, we report a rapidly acting pH‐sensitive fluorophore, **PH08**, to highlight small tumor lesions with high TBR. This novel NIR fluorophore exhibits rapid signal increases in acidic TME by reducing intramolecular quenching.^[^ ^38^, ^39^ ^]^ In addition, to resolve the historical issue of lack of targeting mechanisms of fluorophores, we introduced inherent cancer‐targeting within the compounds' chemical structure, the “Structure Inherent Targeting (SIT)” strategy,^[^ ^40^, ^41^, ^42^, ^43^, ^44^ ^]^ for FGS. By introducing different surface charges and lipophilicity to the backbone of the polymethine structure using various alkyl groups, PH08 showed endogenous affinity to organic anion transporter peptides (OATPs) and enters cancer cells primarily via OATPs and retains in the mitochondria and lysosomes, supporting a long retention time. The most common epithelial ovarian cancer (EOC) histological subtype is high‐grade serous carcinomas (HGSC),^[^ ^45^ ^]^ which account for > 50% of ovarian epithelial malignancies and typically have poor prognoses. HGSC is of clinical importance because it is associated with mutations in the TP53 gene and occasional germline mutations in the BRCA1 and BRCA2 genes.^[^ ^46^ ^]^ Our primary focus is therefore to detect EOC. To this end, we used orthotopic models of ovarian cancer in mice that reasonably mimic the site‐specific pathology of human disease.^[^ ^47^ ^]^ The topical application of the rationally designed **PH08** allows us to visualize small peritoneal dissemination within 10 min without washing, which lasts over 4 h post‐administration, enabling real‐time FGS.

## 2 Results

### 2.1 Systemic Synthesis and Optical Characterization of pH Sensing NIR Fluorophores

**Figure 1:**
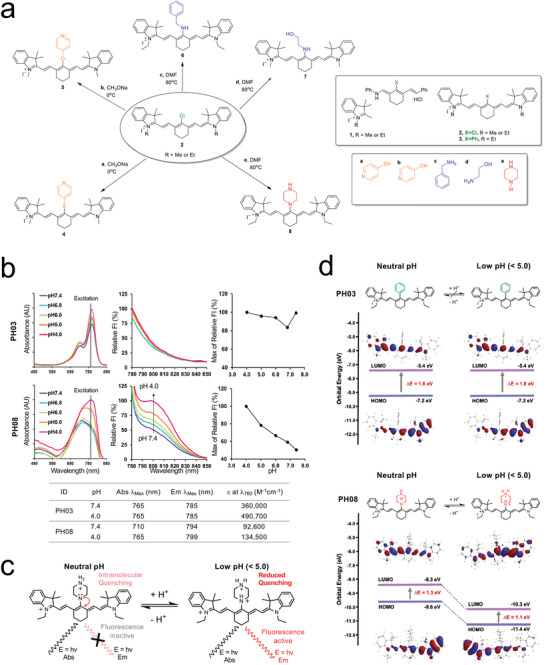
Chemical structures, physicochemical properties, and optical properties of pH sensing NIR fluorophores: a) Chemical structures of pH sensing and non‐sensing NIR fluorophores. b) Optical properties of representative fluorophores. pH‐dependent changes in absorption and fluorescence emission were determined in phosphate‐buffered saline (PBS) with 5% BSA. c) Proposed pH‐dependent changes in electron density distributions and intramolecular quenching of the candidate PH probes. d) Schematic illustration of pH‐dependent intramolecular changes in electron density distributions and HOMO and LUMO energy levels of **PH03** and **PH08** based on DFT calculations. The HOMO and LUMO energy levels were plotted based on the optimized S0 and S1 geometries using Gaussian 16 at B3‐LYP/6‐31G (d).

To achieve rapid and durable intraoperative imaging, we rationally designed activatable NIR fluorophores to detect mildly low pH by placing an amino group near the conjugated double bonds of a cyanine analog, which allows for the delocalization of electrons and the reduction of intramolecular quenching. As shown in **Figure** **1**a, the synthesis of heptamethine cyanines with a rigid cyclohexenyl ring in the polymethine backbone containing a reactive chlorine atom 2 (denoted as **PH02**) or a phenyl ring 3 (**PH03**) at the meso carbon was accomplished using our published synthetic methods.^[^ ^48^, ^49^ ^]^ Then, the functionalized cyanine derivatives (**PH04‐ PH08**) were synthesized via S_NR_1 reaction between the meso‐chloride of **PH02** and various nucleophiles such as aryl thioether, aryl ether, primary aryl amine, alkyl amine, and secondary amine, respectively, all suitable moieties to probe acidic environments.^[^ ^50^ ^]^ For pH‐sensitive probes **PH04** and **PH05,** due to the limited nucleophilicity of protonated thiol and hydroxyl groups, sodium methoxide was introduced as a base at a lower temperature to generate the reactive thiophenoxide and phenoxide ions. For the primary and secondary amine substitutions, due to the increased nucleophilic nature of the amines used, the synthesis of **PH06‐PH08** did not require the use of any bases. The crude products were purified by precipitation using DMSO/diethyl ether or methanol/diethyl ether or by column chromatography using 5–10% methanol in DCM as an eluent to produce **PH04‐ PH08**. Upon measurement of the optical and physicochemical properties of the NIR fluorophores (Table 1), **PH08** was found to be optically favorable for pH sensing of TME (Figure S2, Supporting Information). **PH08** shows a modest molecular absorbance (*ε* at 760 nm = 92 600 M^−1^cm^−1^) at neutral pH condition (pH = 7.4) but increases in absorbance (*ε* at 760 nm = 134 500 M^−1^cm^−1^) accompanying with a red‐shift from 710 to 765 nm when pH decreases to 4.0 (Figure 1b). In contrast, **PH03** displays little to no change in electron density distributions in response to low pH (Figure 1c). Based on these optical properties, we further examined the pH sensing properties of **PH08** while using **PH03** as a control. To understand the effect of changes in electron density distributions in response to pH changes, possible charge states, the optimized molecular structures, and frontier molecular orbitals (FMO) for compounds **PH03** and **PH08** in their deprotonated forms, **PH08^+^** and **PH08^2+^** were calculated based on DFT with 6–311 G basis set (Figure 1d). **PH03** and **PH08** bear a phenyl or a piperazine moiety, respectively, at the central meso position, which significantly impacts the distribution of electrons within the highest occupied molecular orbitals (HOMOs) and the lowest unoccupied molecular orbitals (LUMOs) of these compounds. In the optimized molecular structures, the piperazine moiety of PH08 adopts a chair conformation in both the HOMO and LUMO energy levels.

**Table 1:** Physicochemical and optochemical properties of pH sensing contrast agents

ID	MW [Da]	LogD, pH 7.4	TPSA [Å^2^]	Rot. bonds	Abs *λ*_Max_ [nm]	Em *λ*_Max_ [nm]	*ε* [M^−1^cm^−1^]	QY [%]	TBR
**PH03**	553.81	6.78	6.25	6	765	785	364 800	21.7	++
**PH08**	561.84	2.38	21.52	1	710	794	43 800	16.0	+++

MW, molecular weight; TPSA, total polar surface area; Rot. Bonds, rotatable bonds; *λ*_Max_, maximum wavelength; *ε*, extinction coefficient; QY, quantum yield. Tumor to background ratio (TBR) against normal pancreas was quantified and labeled as −, < 1; +, 1–2; ++, 2–3; +++, 3–5. Measurements were performed at pH 7.4.

We further performed quantum chemical calculations on the protonated forms, **PH08^+^** and **PH08** ^**2** **+**^, using spartan 18. The energy gap decreases from 1.3 to 1.1 eV for **PH08^+^** and **PH08^2+^**, which explains the absorption wavelength change of **PH08** in different pHs at 7.4 and 5.0. There are no distinct differences between the HOMO and LUMO for all the possible species of the optimized molecular structures. Based on the results of the optical property, **PH08** advanced to further in vitro and in vivo testing for cancer imaging.

Next, the optostability of **PH08** was determined by measuring the fluorescence at different concentrations under excitation by a reflectance imaging system in 5% bovine serum albumin (BSA) (Figure S2, Supporting Information). Quenching of **PH08** started at a concentration of 10 µm **PH08** after 760 nm laser irradiation with a 0° geometry intraoperative imaging system (FLARE). The working concentration shifted to a range of 20 to 30 µm, similar to indocyanine green (ICG) (Figure S2a, Supporting Information). Photobleaching curves were also obtained by incubating different concentrations of **PH08** in 5% BSA for 3 h under irradiation with the FLARE system. **PH08** kept at least 50% of its initial fluorescence intensity and was photostable over the incubation time. (Figure S2b, Supporting Information).

### 2.2 Toxicity of pH Sensing NIR Fluorophores

Next, we determined if the pH sensing fluorophore displays any toxicity to cells. To this end, cultured murine ID8 ovarian cancer cells were incubated with various concentrations of **PH08** and then evaluated for cell viability. **PH08** only showed toxicity to ID8 cells at a high concentration of over 3 µm (Figure S3a, Supporting Information). These results indicate that **PH08** can be used safely for imaging.

### 2.3 The Ovarian Cancer Cell Targetability of pH Sensing NIR Fluorophores In Vitro

The ovarian cancer cell targetability of the pH sensing fluorophore **PH08** was determined by fluorescence microscopy using cultured murine ID8 and human SKOV3 and CAOV3 ovarian cancer cell lines, along with NIH3T3 fibroblasts and C2C12 myoblasts used as normal cell controls. **PH08** showed significantly higher accumulation in human ovarian cancer cells than in normal cell lines, although we did not observe a notable difference between murine cancer and normal cell lines in this experimental model (Figure S3b, Supporting Information). These results indicated that **PH08** could be taken up by cells and supported further testing of **PH08** for cancer imaging in vivo.

### 2.4 The Role of Organic Anion Transporters in the Cellular Uptake of NIR Fluorophores

**Figure 2:**
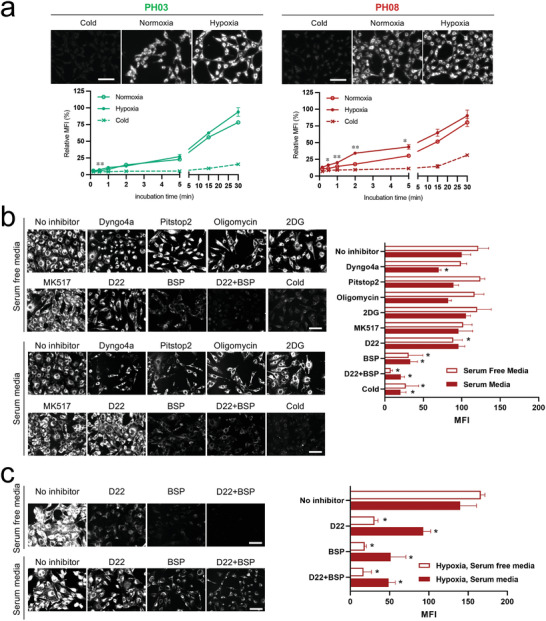
Cellular uptake of pH sensing NIR fluorophores via membrane transporters: a) In vitro tumor cell uptake of the pH sensing NIR probes under normoxic and hypoxic conditions. Murine cancer cell line ID8 cells were incubated in normoxic or hypoxic conditions (1% O_2_) for 24 h at 37 °C, followed by incubation with 0.1 µm NIR probes for 30 min. (a, top) Representative fluorescence images of cells are shown. The contrast was normalized across all images. Scale bar = 100 µm. (a, bottom) Quantitative time‐course measurements of the fluorescence intensity in cells (n = 3, mean ± s.d.). *P < 0.05, **P < 0.01 by two‐way ANOVA followed by Tukey's multiple comparisons test (normoxia vs hypoxia). b) Inhibition assay of cellular uptake of **PH08** to determine the entry mechanisms in ID8 cells. Cultured cells were incubated with bromsulphthalein (BSP), MK‐571, D22, 2DG, oligomycin, Dyngo 4a, or Pitstop 2 for 5–30 min, and then incubated with 0.2 µm **PH08** in 10% or 0% FBS media for 15 min. Alternatively, cells were incubated at 4 °C for 30 min. Cells were then imaged under epifluorescence NIR microscopy. (b, left) Representative fluorescence images of cells are shown. The contrast was normalized across all images. Scale bars = 100 µm. (b, right) Quantitative measurements of the fluorescence intensity in cells (*n* = 3–7, mean ± s.d.). c) Inhibition assay in the hypoxic (1% O_2_) condition. Cultured cells were incubated with BSP, D22, or both, and then incubated with **PH08** in 10% or 0% FBS media. (c, left) Representative fluorescence images of cells are shown. The contrast was normalized across all images. Scale bars = 100 µm. (c, right) Quantitative measurements of the fluorescence intensity in cells (*n* = 3, mean ± s.d.). *P < 0.05 by one‐way ANOVA followed by Tukey's multiple comparisons test.

We next sought the mode of action for cellular uptake of the NIR fluorophores. The uptake mechanism of cyanine‐based, tumor‐targeting heptamethine fluorophores into cancer cells has been proposed to be mediated via OATPs.^[^ ^51^ ^]^ Alternatively, it has also been implicated that serum protein‐bound heptamethine fluorophores accumulate in tumor tissue via the enhanced permeability and retention (EPR) effect and are up‐taken via endocytosis.^[^ ^52^ ^]^ Thus, we hypothesized that the cellular uptake of heptamethine‐fluorophores of **PH08** in cancer tissues was mediated by membrane transporters and endocytosis. The cancer cell uptake of each fluorophore was examined in vitro using ID8 cells (a murine ovarian cancer cell line) by fluorescence microscopy. Both **PH03** and **PH08** accumulated in cells in a time‐dependent manner (**Figure** **2**a).

Cellular uptake, however, significantly decreased when cells were incubated at 4 °C, indicating that these fluorophores hardly enter cells via diffusion and that their entry is dependent on the action of transporters or endocytosis. The organic anion transporting polypeptides (OATPs) is known to mediate the transmembrane uptake of small molecule drugs^[^ ^53^ ^]^ and include structurally related fluorophores such as the clinically approved ICG^[^ ^54^ ^]^ and tumor‐targeting IR‐780.^[^ ^51^ ^]^ Interestingly, the cellular uptake was augmented under hypoxic conditions, which is consistent with the previous observation that OATPs have been established to be upregulated by hypoxia and expressed predominantly in cancer tissues compared with normal tissues.^[^ ^53^ ^]^ Together, this result suggests that **PH08** shows a higher affinity to OATPs than **PH03** with its inherent targeting potency which arises from the chemical structure of compound, representing SIT.^[^ ^40^, ^41^, ^42^, ^43^, ^44^ ^]^

We further determined the possible contribution of other membrane transporters including organic cation transporters (OCTs) and organic carnitine (zwitterion) transporters (OCTNs), which are known to play critical roles in the transcellular movement of a wide variety of xenobiotics, including small organic cationic molecules,^[^ ^55^ ^]^ and may play roles in the uptake of **PH08**. To this end, ID8 ovarian cancer cells were treated with inhibitors for OATPs, OCTs, or OCTNs. We also compared the cellular uptake in serum and serum‐free conditions to determine the involvement of serum proteins and endocytosis. Consistently, the treatment with bromsulphthalein (BSP), which is an OATP inhibitor, resulted in a significant reduction in cellular uptake (Figure 2b, the BSP vs the non‐treated control group: *P* < 0.05 for serum and serum‐free conditions), indicating that **PH08** cellular uptake is mediated by OATPs. The treatment with D22, which is an inhibitor of OCTs and OCTNs, induced a modest inhibitory effect in cellular uptake (Figure 2b, the BSP vs the no inhibitor control group: *P* < 0.05 for serum‐free condition, ns for serum condition), suggesting a minor but important contribution of this membrane transporter. Consistently, the dual inhibition with BSP and D22 further decreased the cellular uptake of **PH08**. MK‐571, an inhibitor for ATP‐binding cassette (ABC) transporters, induced little change in cellular uptake (Figure 2b). Treating cells with Pitstop 2, a clathrin‐dependent endocytosis inhibitor, did not result in any appreciable change in cellular uptake. Dyngo 4a, a dynamin‐dependent endocytosis inhibitor, showed a minor but noticeable inhibitory effect in the serum condition (Figure 2b, the Dyngo 4a vs the no inhibitor control group: *P* < 0.05), suggesting a minor role in endocytosis in the cellular uptake of **PH08** in the presence of serum proteins. We also determined if the balance of the cellular uptake was affected in hypoxic conditions, where OATPs were upregulated.^[^ ^53^ ^]^ Cellular uptake of **PH08** significantly decreased when cells were incubated at 4 °C, confirming that **PH08** hardly entered cells via passive diffusion and was taken up by transporters. Based on these results, we further determined the role of OATPs and OCTs in hypoxic conditions. BSP showed the most prominent inhibition effect (Figure 2c, the BSP vs the no inhibitor control group: *P* < 0.05 for serum and serum‐free conditions), although the significant, but smaller inhibition from D22 was observed for serum and serum‐free conditions under the hypoxic condition compared to BSP (Figure 2c, the D22 vs the no inhibitor control group: *P* < 0.05 for serum and serum‐free conditions). The dual inhibition with BSP and D22 did not further decrease the cellular uptake of **PH08**, suggesting the predominant role of OATPs in hypoxic conditions.

To enhance the translatability of this technology, we further determined whether human ovarian cancer had equivalent mechanisms of action of the cellular uptake. To this end, we tested the cellular uptake of **PH08** in human ovarian cancer cell line SKOV3. Consistent with the results in the murine cell line, BSP showed a significant inhibitory effect in both normoxic and hypoxic conditions (Figure S4, Supporting Information, the BSP vs the no inhibitor control group: *P* < 0.05 for serum and serum‐free conditions), demonstrating that OATP predominantly mediated the cellular uptake of **PH08**. The moderate inhibition with D22 was observed in the serum and serum‐free conditions under normoxic and hypoxic conditions (Figure S4, Supporting Information, the D22 vs the no inhibitor control group: *P* < 0.05 for serum and serum‐free conditions). The dual inhibition with BSP and D22 further decreased the cellular uptake of PH08 (Figure S4, Supporting Information), demonstrating the predominant role of OATPs and the significant contribution of OCTs in the cellular uptake of PH08.

### 2.5 Subcellular Distribution of pH Sensing NIR Fluorophores

**Figure 3:**
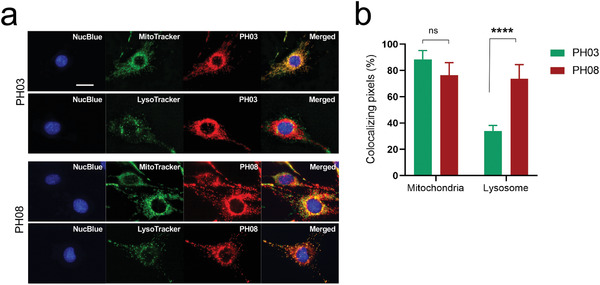
Subcellular localization of pH sensing NIR fluorophores: a) Subcellular localization of each probe (10 µm) in ID8 cells was determined after 30 min of incubation at 37 °C. Nuclei, mitochondria, and lysosomes were stained with NucBlue, MitoTracker Green, or LysoTracker Red, respectively. Representative fluorescence images of cells. Scale bar = 20 µm. b) Co‐localization index of each probe with MitoTracker Green or LysoTracker Red. The index was calculated by dividing the area of overlap between pH probe and MitoTracker or LysoTracker by the total area of MitoTracker or LysoTracker, respectively. The co‐localization index was determined in 5–6 photographic areas (70 × 92 µm^2^ each) for each probe. *P < 0.05 by two‐way ANOVA followed by Tukey's multiple comparisons test. Error bars show means ± s.d.

To investigate the fate of the NIR fluorophores after uptake by cells, we performed in vitro live‐cell imaging and evaluated the subcellular localization of the NIR fluorophores in cultured ID8 ovarian cancer cells. **PH03** or **PH08** was incubated with ID8 cells for 30 min followed by double staining with commercially available MitoTracker or LysoTracker Green and imaged under the house‐built NIR fluorescence microscope. NucBlue staining confirmed cell viability throughout the experiment. The fluorescence signal of **PH03** overlapped with the signal of MitoTracker (91.47%), but was significantly less than that of LysoTracker (34.93%) (**Figure** **3**a). On the other hand, **PH08** showed much higher signals in the lysosome (71.83%), while maintaining similar signals in the mitochondria (83.05%) compared with **PH03** (Figure 3b). No signal was observed in nuclei for both NIR fluorophores. This is consistent with previous observations that lipophilic cations, including cyanine derivatives, accumulate in mitochondria^[^ ^56^ ^]^ and lysosomes.^[^ ^57^ ^]^ Together, these results suggest that **PH08** enters cells via transporters and is well retained in cellular organelles.

### 2.6 Evaluation of In Vivo Efficacy of pH Sensing NIR Fluorophores for Fluorescence‐Guided Surgery (FGS)

**Figure 4:**
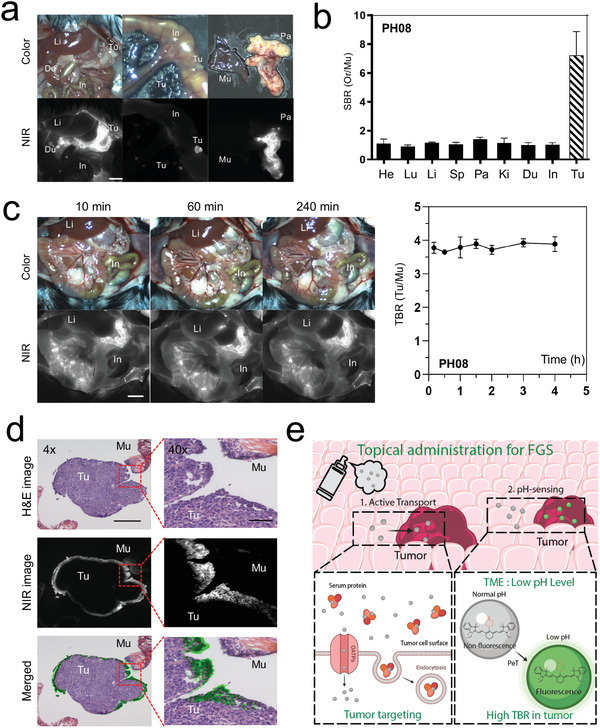
In vivo evaluation of tumor cell targetability of pH sensing NIR fluorophores in a mouse model of ovarian cancer: a) Intraoperative color and NIR fluorescence images of the abdominal cavity and peritoneal dissemination of ovarian cancer and major organs 4 h post‐intraperitoneal injection of 20 nmol of **PH08**. Scale bar = 5 mm. b) Quantitative analysis of TBR of peritoneal tumors and major organs for each NIR fluorophore. TBR was determined by comparing the signals of tumors (Tu) or organs against muscle (Mu). Du, duodenum; He, heart; In, intestine; Ki, kidneys; Li, liver; Lu, lungs; Pa, pancreas; Sp, spleen; St, stomach (*n* = 3, mean ± s.e.m.). c) Quantitative time‐course assessment of TBR for up to 240 min post‐injection (*n* = 3, mean ± s.e.m.). d) Histological analysis of tumor‐targeted **PH08** in an orthotopic ovarian cancer model. Representative hematoxylin and eosin (H&E) and corresponding NIR fluorescence images are shown. Mu, muscle; Tu, tumor. Left and right panels: scale bars = 500 and 50 µm, respectively. e) A summary schematic of the study. PH08 is taken up by tumor cells via OATPs and endocytosis and retained in organelles upon topical application. The change in electron density distributions and rapid reduction in intramolecular quenching occurs in the acidic TME and lysosomes of cancer cells, where **PH08** fluorescence emission is enhanced, realizing high TBR of small peritoneal dissemination of ovarian cancer during optical imaging for a sufficiently long time.

Having confirmed the pH sensitivity and tumor cell uptake of **PH08** in vitro, we next evaluated the in vivo performance of the pH sensing NIR fluorophores in a mouse model of ovarian cancer. The intraperitoneal administration of an ID8 murine ovarian cancer cell line has been shown to closely mimic human advanced‐stage ovarian cancer and its histology.^[^ ^58^ ^]^ In this model, approximately 12 wk post‐peritoneal injection of ID8 cells, numerous nodules were observed on the peritoneum. These small nodules (< 1 mm) are extremely difficult to localize and visualize in the surgical field. Thus, this model was suitable to test the performance of this fluorophore. For imaging of ovarian cancer, 20 nmol of **PH08** was administered intraperitoneally. The abdominal cavity was then imaged on the custom‐built imaging system.^[^ ^10^, ^59^ ^]^ Within 10 min of **PH08** injection, small tumors planted on the peritoneal surface and omentum were clearly visualized (**Figure** **4**a) with a high TBR (≈6) against the surrounding normal tissues and other major organs due to the ultralow background (Figure 4b). The signal of PH08 allowed for clear visualization of the lesions that measured 5 mm or less than 1 mm in diameter. The TBR stayed high (> 5.5) up to 4 h after injection (**Figure** **4c**). The pH insensitive NIR fluorophore **PH03** also showed fluorescence signals in tumor tissue but displayed high signals in the normal tissue as well (Figure S5a,b, Supporting Information). Furthermore, due to the relatively high hydrophilicity of this fluorophore, the background signal remained high up to 4 h after administration with smaller TBRs (Figure S5c,d, Supporting Information). PH08 did not distribute to the major organs including vital tissues (Figure S5e, Supporting Information), indicating little to no safety concerns. Taken together, the topical application of **PH08** with a low dose (≤20 nmol) avoids unexpected off‐target accumulation in vital organs, which reduces the risks of adverse effects.

Having confirmed the in vivo efficacy of **PH08**, we performed FGS 4 h post‐injection. The NIR imaging provided precise localization of peritoneal metastatic nodules and enabled the surgeon to achieve complete resection of metastatic nodules, including a small nodule measuring < 1 mm in size (Movie S1, Supporting Information).

In order to validate the tumor‐specific fluorescence signal, frozen sections including both tumor and adjacent normal tissues harvested from tumor‐bearing mice were examined by fluorescence microscopy. Histologically, the strong fluorescence signals of **PH08** were detected throughout the tumor tissue with no substantial fluorescence signals in neighboring muscles, showing a clear contrast between tumors and surrounding normal tissues (Figure 4c). The fluorescence signal was mostly seen on the top 3–4 layers of cells in the tumor tissue, of which the signal was found predominantly in the extracellular space and cytoplasm. There was a clear distinction in fluorescence intensity between cancerous and normal tissue, and no fluorescence signal was observed in the peritoneal surface nearby or in other surrounding normal tissues. These results suggest that the low molecular weight of **PH08**, along with its lipophilicity, supported efficient diffusion into tumor tissue, and its pH sensitivity helped achieve sufficiently high TBR for FGS. Consistently, **PH08** was able to clearly visualize small human ovarian tumors (SKOV3) planted on the omentum in a xenograft model (Figure S5f,g, Supporting Information) with no substantial fluorescence signals in the neighboring normal tissue (Figure S5h, Supporting Information). Together, these results suggest that this novel pH sensing NIR fluorophore is well suited for FGS and would merit advanced preclinical development.

## 3 Discussion

In this study, we have successfully developed a tumor‐targeted contrast agent, **PH08**, which is rationally designed for pH‐sensing. In addition, **PH08** shows an excellent affinity to OATPs, enters cancer cells via these membrane transporters, and shows excellent retention in the mitochondria and lysosomes. With these two mechanisms, **PH08** can detect submillimeter peritoneal dissemination of ovarian cancer within 10 min of topical application with high TBR, and its specific fluorescence signal in tumor lesions was observed under NIR imaging up to 4 h. **PH08** has overcome historical shortcomings of activatable NIR fluorophores, including no tissue targetability, long staining time, short retention time, and low TBR. **PH08** shows favorable optical properties and increases its fluorescence in mildly acidic conditions between pH 7.0 and 4.0, contributing to its rapid staining of tumor tissue. Most pH sensing probes that have been developed to date only display PeT at relatively high pH values.^[^ ^60^ ^]^ Unlike other pH sensing probes, our rationally designed probe shows changes in electron density distributions in mildly acidic conditions and rapid reduction in intramolecular quenching, which favors specific detection of TME. In addition, **PH08** shows inherent targeting potency for OATPs, which are overexpressed in cancer, and excellent tumor‐targetability. A low molecular weight without the need for attachment of a targeting moiety is a critical property of **PH08** because it enables local application, which passes through the acidic TME and highlights tumor margins with high TBR. These physicochemical properties had combinatorial effects on the TBR. This NIR fluorophore is therefore optimal for image‐guided biopsy of superficial tumors, including colon and cervical cancer. More is known about the toxicity of polymethine fluorophores than virtually any molecule ever administered to humans because ICG, which contains the same backbone as **PH08**, has shown limited adverse reactions.^[^ ^61^ ^]^ In addition, there is extensive animal toxicology data^[^ ^12^ ^]^on dozens of other NIR fluorophores, which proves that the doses we propose to use in humans are orders of magnitude lower than toxic levels. Additionally, the proposed administration route for **PH08** is to topically spray onto tissue surfaces that are suspected to harbor tumors; requiring about a tenfold lower dose than systemic administration.^[^ ^62^, ^63^, ^64^ ^]^ In addition, since **PH08** shows durable retention in organelles, its fluorescence signal is stable for at least 4 h after application. With this feature, it can also be used for laparoscopic or open surgery of peritoneal dissemination of ovarian and gastrointestinal cancer. Such an easy‐to‐use, economical and reliable tool is expected to have a significant impact on surgical care because image‐guided robotic or laparoscopic surgery in the form of FGS is expected to be the predominant mode of surgery in the following decades.^[^ ^65^ ^]^

Previously, other strategies have been used for the design of “activatable” probes to image tumor tissue. Generally, the activation process of these molecular probes requires hours to even days, which decreases the feasibility of this approach and chances for real‐time imaging.^[^ ^29^ ^]^ The targeting of enzymatic activity, whose expression is elevated in tumor tissues, has the potential to achieve rapid detection of cancer. Cathepsin and MMP‐activatable probes are based on the unquenching of stacked fluorophores; therefore, their fluorescent intensity upon activation is not sufficiently high to detect small cancer lesions.^[^ ^66^ ^]^ Topically applied activatable fluorophores are activated by the membrane‐bound enzyme *γ*‐glutamyltranspeptidase (GGT), which is overexpressed on the plasma membrane of tumor cells, enabling the fluorescent detection of small‐sized tumors for up to 1 h post‐injection.^[^ ^63^ ^]^ However, this method is still limited due to the complicated and large structure of the probe complex and the lack of scalability for clinical use. **PH08** has the clear advantages of smaller molecular weight and simple chemical structure, which significantly reduces the production labor and cost.

The use of a small molecule as a contrast‐enhancing agent has significant advantages in targeted delivery to tumor tissue because uptake of small molecules mainly relies on diffusion rather than convection.^[^ ^67^ ^]^ However, it has been challenging to obtain sufficiently high TBR using small molecule contrast‐enhancing agents due to their rapid clearance, high background signal, and low target affinity.^[^ ^68^ ^]^ A small clinical trial shows the efficacy of an FDA‐approved small molecule fluorophore ICG for FGS of ovarian cancer.^[^ ^69^ ^]^ In this study, due to the weak targetability of ICG, a high false‐positive rate was reported. In the current study, both **PH03** and **PH08** were able to target ovarian cancer tissue and visualize the lesions (Figure 4). Without an activation mechanism, however, “always‐on” probes (e.g., **PH03**) rely on their targetability to specific tissues, which generally results in low TBR due to their elevated uptake in the background tissue unless they are designed for rapid renal clearance. In contrast, “activatable” **PH08** with the same targeting mechanism was able to achieve high TBR due to the low background signal. Since pH sensitivity contributes an approximate 50% increase in fluorescence signal, the significant improvement of **PH08**'s TBR compared to that of **PH03** may be explained by the improved cellular uptake of **PH08** due to the lysosomal sequestration of protonated lipophilic amines at low pH. This concept of using a “smart” small molecule with intrinsic targetability and activation mechanisms should be considered for the future design of molecular probes for cancer imaging and following FGS. Generally, the small size of the tracer is advantageous for tissue penetration. The tissue penetration was also affected by the physicochemical properties of the dye, including charge and hydrophobicity. Although the size is small, the tissue penetration of **PH08** was 3–4 cell layers deep (Figure 4e). Further optimization of **PH08** is warranted to achieve deep penetration by optimizing the physicochemical properties of this lipophilic cation.

In conclusion, the rationally designed activatable **PH08**, which shows a reduction in intramolecular quenching in TME, and cancer cell targetability via OATPs shows rapid fluorescence recovery (< 10 min) upon topical application and durable retention in the mitochondria and lysosomes of tumor cells (at least 4 h post‐topical application). **PH08** has the potential to reduce operation times and decrease the likelihood of residual tumors after surgery. Furthermore, since **PH08** is a small molecule, it provides a scalable strategy for production and has a high potential for accelerated approval by the FDA for clinical use of tumor targeting, following the safety and regulatory guidelines of ICG.

## 4 Experimental Section

### Chemicals and Synthesis

All chemicals and solvents were of American Chemical Society grade or HPLC purity and were used as received. HPLC grade acetonitrile (CH_3_CN) and water were purchased from VWR International (West Chester, PA) and American Bioanalytic (Natick, MA), respectively. All other chemicals were purchased from Fisher Scientific (Pittsburgh, PA) and Sigma‐Aldrich (Saint Louis, MO), unless otherwise noted. Melting points were measured on a Meltemp apparatus and were uncorrected. ^1^H‐ and ^13^C‐NMR spectra were recorded on a BrukerAvance (400 MHz) spectrometer. Vis/NIR absorption spectra were recorded on a Perkin Elmer Lambda 20 spectrophotometer or Varian 50 scan UV‐visible spectrophotometer. Chemical purity was also confirmed using high‐performance liquid chromatography (HPLC, Waters, Milford, MA) combined with simultaneous evaporative light scattering detection (ELSD), absorbance (photodiode array; PDA), fluorescence, and electrospray time‐of‐flight (ES‐TOF) mass spectrometry (MS). High‐resolution accurate mass spectra (HRMS) were obtained either using a Waters ESI‐Q‐TOF mass spectrometer or a Waters Micromass LCT TOF ES^+^ Premier Mass Spectrometer. The detailed methods are described in Supporting Information. All fluorophores were prepared as 10 mm stock solutions in dimethylsulfoxide (DMSO) prior to use.

### Measurement of Optophysical Properties

Absorbance and fluorescence emission spectra of the series of NIR fluorophores were measured using fiber optic HR2000 absorbance (200–1100 nm) and USB2000FL fluorescence (350–1000 nm) spectrometers (Ocean Optics, Dunedin, FL). NIR excitation was provided by a 655 nm red laser pointer (Opcom Inc., Xiamen, China) set to 5 mW and coupled through a 300 µm core diameter, NA 0.22 fiber (Fiberguide Industries, Stirling, NJ). To determine the pH dependence of the fluorescence signal, optical measurements were performed at 37 °C in PBS, pH 4.0–7.4, or 100% fetal bovine serum (FBS) buffered with 50 mm HEPES, pH 4.0–7.4. The pH of the solutions was adjusted as appropriate with NaOH and HCl using a pH spear tester (Thermo Scientific). For fluorescence quantum yield (QY) measurements, ICG in DMSO (QY = 13%) was used as a calibration standard to measure the fluorescence QY under conditions of matched absorbance at 765 nm.^[^ ^70^ ^]^ In silico calculations of the partition coefficient (Log*P* and Log*D* at pH 7.4), surface molecular charge, hydrophobicity, hydrogen bond acceptors/donors (HBA/HBD), the acid dissociation constant (pKa), and topological polar surface area (TPSA) were calculated using Marvin and JChem calculator plugins (ChemAxon, Budapest, Hungary). Theoretical calculations of HOMO and LUMO energy levels of fluorophores were performed based on density functional theory (DFT) with a 6–311 G basis set using Spartan 18 software (Wavefunction, Irvine, CA).

### Stability Studies of Squaraine Fluorophores

Stability experiments were performed to determine the photobleaching of the fluorophores. This was determined by measuring the absorbance at 760 nm over a 3 h time period. Measurements were taken intermittently at indicated time points. The light exposure condition involved a custom‐built real‐time intraoperative NIR imaging system.^[^ ^71^ ^]^ A cuvette containing an individual contrast agent (5, 10, 15, or 25 µm) in 5% BSA solution was placed 15 cm away from the light source and exposed to 760‐nm laser excitation at a fluence rate of 2 mW cm^−2^ at room temperature. The integrated absorbance from the maximum absorption wavelength (634 nm for 5% BSA) to the baseline was determined at each indicated time point.

### Live‐Cell Labeling and In Vitro Imaging

Human (SKOV3, CAOV3) and murine ovarian cancer cells (ID8 cells) were obtained from Dr. Wang's lab in the Vincent Center for Reproductive Biology at Massachusetts General Hospital (MGH). NIH/3T3 murine fibroblast cell line (CRL‐1658) and C2C12 murine myoblast cell line (CRL‐1772) were purchased from ATCC (Manassas, VA). Cells were cultured and maintained in Dulbecco's modified Eagle's medium (DMEM) supplemented with 4% FBS (Gibco, Grand Island, NY), 1% insulin‐transferrin‐selenium (Sigma‐Aldrich), and 1% penicillin‐streptomycin (Gibco) or DMEM supplemented with 10% FBS, 1% penicillin/streptomycin (for NIH/3T3 and C2C12 and CAOV3), or McCoy's 5a modified medium supplemented with 10% FBS (for SKOV3) at 37 °C in a humidified 5% CO_2_ atmosphere. Cells were plated in a T‐75 culture flask until 70% confluency before dividing for the next passage.

For fluorophore binding affinity, ID8, SKOV3, CAOV3, C2C12, and NIH/3T3 cells were incubated at a density of 20 000 cells/well in a 24‐well plate in a humidified incubator at 37 °C under 5% CO_2_ in the air. Cells were then incubated at 37 °C for 30 min in the presence of 0.2 µm of **PH03** or **PH08** and phenol red‐free Hank's balanced salt solution (HBSS) with or without 10% FBS. To determine the response of NIR fluorophores to hypoxic conditions, cells were also incubated at 37 ℃ in a humidified incubator under 21% O_2_ and 5% CO_2_ (normoxia) or 1% O_2_ and 5% CO_2_ (hypoxia) conditions for 24 h. The hypoxic condition was created using a hypoxic chamber (Coy Laboratory Products) with 5% CO_2_ / 95% N_2_ (Airgas, Lynn, MA). After washing three times with HBSS, live‐cell imaging was performed using BioTek Cytation 5 (Winooski, VT) and NanoenTek JuLI Stage Live‐Cell Imaging System (Seoul, S. Korea) with a Cy7 filter set (Chroma Technology, Brattleboro, VT). Image acquisition and analysis were performed using IPLab software (Scanalytics, Fairfax, VA), and the fluorescent intensity of each cell was measured using ImageJ (National Institutes of Health, Bethesda, MD). The outline of each cell was determined on a phase‐contrast image to specify the region of interest (ROI) and superimposed on a fluorescence image for measurements. All NIR fluorescence images for each fluorophore were normalized identically for all conditions during an experiment.

To determine the subcellular localization of NIR fluorophores, cells were seeded on a glass‐bottomed dish (µ‐Dish 35 mm low; Ibidi, Gräfelfing, Germany) at a density of 10 000 cells per dish and allowed to adhere overnight and then incubated in growth media with the presence of **PH03** or **PH08** at a concentration of 10 µm for 30 min. After being washed three times with HBSS, nuclei, mitochondria, and lysosomes were then stained with NucBlue (2 drops mL^−1^), MitoTracker Green FM (0.5 µm), or LysoTracker Green (0.5 µm) for 20–30 min as per the manufacturer's instruction (Thermo Scientific, Waltham, MA). Cells were then washed with HBSS and imaged using the 4‐channel Nikon TE2000 epifluorescence microscope. The co‐localization of NIR fluorophores and organelles of interest was assessed by Mander's colocalization coefficients using Costes method on ImageJ.^[^ ^72^ ^]^ Threshold values were optimized as previously described.^[^ ^73^ ^]^

### Cell Viability Assay

ID8 cells were plated onto 96‐well plates at a density of 5 × 10^3^ cells per well. Cells were then treated with 0–25 µm of **PH08** in growth media for 24 h. Cell viability was determined using the Cell Counting Kit‐8 (CCK‐8) from Dojindo Molecular Technologies, Inc. (Kumamoto, Japan). Briefly, 10 µL CCK‐8 was added to each well, and the plates were incubated for 3 h at 37 °C. Absorbance was measured at 450 nm using a SpectraMax M5 (Molecular Devices). Survival rate was calculated as follows: Survival rate (%) = (Asample–Ab)/(Ac−Ab) × 100, where Asample, Ab, and Ac denote the absorbance reading of sample, blanks, and negative control wells, respectively.

### Intraoperative NIR Imaging

Seven‐week‐old female C57BL/6 mice were purchased from the Jackson Laboratory (Bar Harbor, ME) and housed in an AAALAC‐certified facility at Massachusetts General Hospital (MGH). All animal procedures were performed in accordance with the Public Health Service Policy on Humane Care of Laboratory Animals and approved by the MGH IACUC (protocol #2016N000136). To establish a murine model of peritoneal dissemination of ovarian cancer, mice were injected intraperitoneally with 5×10^6^ cells in 100 µL saline. In this model, macroscopic metastatic intraperitoneal nodules appeared reproducibly at approximately 90 d. To perform NIR fluorescence imaging, mice were fed chlorophyll‐free mouse chow (VWR International) at least 5 d prior to imaging to minimize autofluorescence. For in vivo imaging of ovarian cancer, animals were anesthetized with 100 mg kg^−1^ ketamine and 10 mg kg^−1^ xylazine subcutaneously (Webster Veterinary, Fort Devens, MA). Following anesthesia, a midline incision was made to expose the abdominal cavity. To evaluate in vivo tumor targetability of the NIR fluorophores, mice were intraperitoneally injected with 20 nmol of ovarian cancer‐targeted NIR fluorophores dissolved in 100 µL saline containing 5 wt/v% BSA. The abdominal cavity was opened with a midline incision, and NIR images were obtained over 4 h of the exposed abdominal cavity. For NIR fluorescence imaging, a custom‐built real‐time intraoperative NIR imaging system (the FLARE imaging system) was used as described previously.^[^ ^8^, ^59^ ^]^ In this study, a 760 nm excitation was used at a fluence rate of 4 mW cm^−2^, with white light (400–650 nm; 40 000 lux). Color and NIR fluorescence images were acquired simultaneously with custom software at rates up to 15 Hz over a 10 cm diameter field‐of‐view (FOV). Pseudo‐colored lime green was used for NIR fluorescence in the color‐NIR merged images. The imaging system was positioned at a distance of 9 inches from the surgical field. For all real‐time intraoperative imaging, a standardized imaging protocol was used during and after the operation. General FOV (5 cm in dia.) was used to include the pancreas, head, duodenum, liver, and kidneys of a mouse, while closeup FOV (3.3 cm in dia.) included the pancreas, head, and duodenum. Color and NIR fluorescence images were taken simultaneously. Real‐time fluorescence signal in tumors (Tu) and TBR compared to neighboring tissue was obtained over the period of imaging, and the fluorescence intensity was plotted to evaluate in vivo molecular biodistribution and clearance. All images were obtained using the same exposure time. Mice were euthanized 4 h post‐injection with CO_2_ inhalation, and tumor tissues and the major organs, including the heart, lung, liver, pancreas, spleen, kidney, duodenum, intestine, muscle, and adnexa, were removed for ex vivo imaging and histological examination. NIR signals from resected tissues were imaged using the FLARE imaging system. The TBR was calculated using the same formula, with *I*_T_ representing the intensity of the tumor tissue and *I*_B_ representing the signal intensity of the surrounding tissue over the imaging period.

### Preclinical Intraoperative Fluorescence‐Guided Surgery

Preclinical FGS was performed in mice with peritoneal dissemination of ID8 ovarian cancer using the FLARE imaging system. 20 nmol of **PH08** was injected in 100 µL of 5% wt/v BSA/saline into the mouse intraperitoneally 4 h prior to the surgery. The abdominal cavity was opened with a midline incision under isoflurane inhalational anesthesia. The entire abdominal cavity was examined carefully by a surgeon using color and fluorescent imaging on the FLARE imaging system. Peritoneal dissemination was resected under white light assisted with the color and fluorescent images.

### Histological Analysis of Tumor‐Targetability of the NIR Fluorophore

To determine the tissue distribution of the NIR fluorophore, tumors and major organs were removed from ID8 ovarian cancer‐bearing mice 4 h post‐injection with 20 nmol of **PH08** in 100 µL of 5% wt/v BSA/saline. The dissected tissues were trimmed and embedded in Tissue‐Tek optimum cutting temperature (OCT) compound (Sakura Finetek, Torrance, CA) without a pre‐fixation step, and the tissue block was frozen at −80 °C. Ten‐µm thick frozen sections were cut by a cryostat (Leica, Germany). The slides were subject to fluorescence analysis first, then stained for hematoxylin and eosin (H&E). Fluorescence and brightfield images were acquired on the 4‐channel Nikon TE2000 epifluorescence microscope. Image acquisition and analysis were performed using IPLab software (Scanalytics, Fairfax, VA). A custom filter set (Chroma Technology, Brattleboro, VT) composed of a 650/45 nm excitation filter, a 685 nm dichroic mirror, and a 720/60 nm emission filter was used for imaging. Exposure times were adjusted to obtain a similar maximum fluorescence value for each fluorescence image. Brightfield images of H&E‐stained slides from a matching field of view were also obtained.

### Statistical Analysis

Statistical analysis was carried out using a one‐way ANOVA followed by Tukey's multiple comparisons test. *P* values less than 0.05 were considered significant: *P <0.05, **P <0.01, and ***P <0.001. Results were presented as mean ± standard errors of the mean (s.e.m.) for all the image analyses on the FLARE system and fluorescence microscopy. Statistical analysis and curve fitting were performed using Microsoft Excel and Prism version 8 software (GraphPad, San Diego, CA).

## Conflict of Interest

The authors declare no conflict of interest.

## Author Contributions

S.Y., T.F., H.M., Y.B., J.K., A.D.U., E.R.B., and S.K. performed the experiments. M.H., H.W., H.K., and K.B. performed chemical synthesis of the fluorophores. S.Y., T.F., H.M., H.W., E.R.B., Y.B., K.I., M.F., X.L., C.W., S.K., and H.S.C. reviewed, analyzed, and interpreted the data. S.Y., E.R.B., M.H., S.K., and H.S.C. wrote the paper. All authors discussed the results and commented on the manuscript.

## Supporting information

Supporting Information

Supporting Movie 1

## Data Availability

The data that support the findings of this study are available in the supplementary material of this article.
