# Supplementary material for: Topical pH Sensing NIR Fluorophores for Intraoperative Imaging and Surgery of Disseminated Ovarian Cancer
Source: Adv Sci (Weinh). 2022 May 14;9(20):2201416. doi: 10.1002/advs.202201416 (PMC9286000; doi:10.1002/advs.202201416)
Supplement: Supplementary file 1 — Supporting Information [file ADVS-9-2201416-s002.pdf]

ADVANCED SCIENCE
Open Access

Supporting Information

for *Adv. Sci.*, DOI 10.1002/advs.202201416

Topical pH Sensing NIR Fluorophores for Intraoperative Imaging and Surgery of Disseminated Ovarian Cancer

Shinya Yokomizo, Maged Henary, Emmanuel R. Buabeng, Takeshi Fukuda, Hailey Monaco, Yoonji Baek, Sophia Manganiello, Haoran Wang, Jo Kubota, Amy Daniel Ulumben, Xiangmin Lv, Cheng Wang, Kazumasa Inoue, Masahiro Fukushi, Homan Kang, Kai Bao, Satoshi Kashiwagi* and Hak Soo Choi**

SUPPORTING INFORMATION

Topical pH Sensing NIR Fluorophores for Intraoperative Imaging and Surgery of Disseminated Ovarian Cancer

Shinya Yokomizo[1,2], Maged Henary[3**], Emmanuel R. Buabeng[3], Takeshi Fukuda[1,4], Hailey Monaco[1], Yoonji Baek[1], Sophia Manganiello[1], Haoran Wang[1], Jo Kubota[1], Amy Daniel Ulumben[1], Xiangmin Lv[5], Cheng Wang[5], Kazumasa Inoue[2], Masahiro Fukushi[2], Homan Kang[1], Kai Bao [1], Satoshi Kashiwagi[1**] and Hak Soo Choi[1**]

[1]Gordon Center for Medical Imaging, Department of Radiology, Massachusetts General Hospital and Harvard Medical School, Boston, MA 02114, USA
[2]Department of Radiological Sciences, Tokyo Metropolitan University, 7-2-10 Higashi-Ogu, Arakawa, Tokyo 116-8551, Japan
[3]Department of Chemistry and Center for Diagnostics and Therapeutics, 100 Piedmont Avenue SE, Georgia State University, Atlanta, GA 30303, USA
[4]Department of Obstetrics and Gynecology, Osaka City University Graduate School of Medicine, 1-4-3, Asahimachi, Abeno-ku, Osaka, 545-8585, Japan
[5]Vincent Center for Reproductive Biology, Vincent Department of Obstetrics and Gynecology, Massachusetts General Hospital, Boston, MA 02114, USA

**Corresponding Authors: M.H. mhenary1@gus.edu; H.S.C. hchoi12@mgh.harvard.edu; or S.K. skashiwagi@ mgh.harvard.edu

TABLE OF CONTENTS

Supplementary Methods

Figure S1. Chemical analyses of PH08 including ^{1}H and ^{13}C NMR, HPLC, and TOF-MS.

Figure S2. Optical properties of candidate fluorophores.

Figure S3. Tumor cell targetability and cytotoxicity of the pH sensing NIR fluorophore.

Figure S4. Cellular uptake of pH sensing NIR fluorophores via membrane transporters in a human ovarian cancer cell line.

Figure S5. *In vivo* evaluation of tumor cell targetability of pH probes in a mouse model of ovarian cancer.

Supplementary Movie S1. Preclinical Fluorescence Guided Surgery.

Supplementary Methods

As shown in **Figure 1a**, the synthesis of heptamethine cyanines with a rigid cyclohexenyl ring in the polymethine backbone containing a reactive chlorine atom **2** or a phenyl ring **3** at the meso carbon was accomplished using our published synthetic methods.[1,2] Then, we followed our published procedure[3] to synthesize the functionalized cyanine derivatives **4-8** *via* $S_{NR}1$ reaction between the meso-chloride of PH02 and various nucleophiles such as aryl thioether, aryl ether, primary aryl amine, alkyl amine, and secondary amine respectively, all suitable moieties to probe the acidic environments. For pH-sensitive probes PH04 and PH05, due to the limited nucleophilicity of protonated thiol and hydroxyl groups, sodium methoxide was introduced as a base at a lower temperature to generate the reactive thiophenoxide and phenoxide ions.[3] Then, the more nucleophilic thiolate or phenoxide ion was allowed to react with dye **2** at room temperature to produce the pH-sensitive probes PH04 and PH05.[3] For the primary and secondary amine substitutions, due to the increased nucleophilic nature of the amines used, the synthesis of pH probes **6-8** (PH06-08) did not require the use of any bases. Therefore, various amines can react with PH02 in DMF without any base at room temperature or through moderate heating of the reaction mixture to furnish the final probes. The crude products of **Fig. 1a** were purified by precipitation using DMSO/diethyl ether or methanol/diethyl ether or by column chromatography using 5-10% methanol in DCM as an eluent to produce the pH-sensitive PH04-08 in good yields.

1,2,3,3-Tetramethyl-3H-indole-1-ium iodide salt (**1**; *R = Me or Et)* and *2-((E)-2-((E)-2-chloro-3-(2-((E)-1,3,3-trimethylinden-2-ylidene)ethylidene)cyclohex-1-en-1-yl)vinyl)-1,3,3-trimethyl-3H-indol-1-ium iodide* (**2**; *R = Me or Et)* were prepared based on our published procedures.[1,2]

1-Ethyl-2-((E)-2-((E)-6-(2-((E)-1-ethyl-3,3-dimethylindolin-2-ylidene)ethylidene)-3,4,5,6-tetrahydro-[1,1'-biphenyl]-2-yl)vinyl)-3,3-dimethyl-3H-indol-1-ium iodide (**3**, *R = Et)* and *1,3,3-trimethyl-2-((E)-2-((E)-2-((E)-2-(pyridine-4-ylthio)-3-((E)-2-(1,3,3-trimethylindolin-2-ylidene)ethylidene)cyclohex-1-en-1-yl)vinyl)-3H-indol-1-ium iodide* (**4**) were prepared based on our published procedures [1,2] and [3], *respectively.*

*1,3,3-trimethyl-2-((E)-2-((E)-2-((E)-2-(pyridine-4-yloxy)-3-((E)-2-(1,3,3-trimethylindolin-2-ylidene)ethylidene)cyclohex-1-en-1-yl)vinyl)-3H-indol-1-ium iodide (***5***), 2-((E)-2((E)-2-*

(benzylamino)-3-(2-((E)-1-ethyl-3,3-dimethylindolin-2-ylidene)ethylidene)-cyclohex-1-en-1-yl)vinyl)-1-ethyl-3,3-dimethyl-3H-1-indol-1-ium, iodide (**6**), and *2-((E)-2-((E)-2-((2-hydroxyethyl)amino)-3-(2-((Z)-1,1,3-trimethyl-1,3-dihydro-2H-inden-2-ylidene)ethylidene)cyclohex-1-en-1-yl)vinyl)-1,3,3-trimethyl-3H-indol-1-ium iodide* (**7**) were prepared according to the previously reported procedures.[3]

2-((E)-2-((E)-3-(2-((E)-1-ethyl-3,3-dimethylindolin-2-ylidene)ethylidene)-2-(piperazin-1-yl)cyclohex-1-en-1-yl)vinyl)-3,3-dimethyl-3H- *indol-1-ium iodide* (**8**). Chlorocyanine dye **2** (860 mg, 1.5 mmol) and piperazine (480 mg, 6 mmol) were dissolved in anhydrous DMF (15 mL) and stirred at 80°C for 2 h under a nitrogen atmosphere. The solvent was removed under reduced pressure, and the resulting residue was purified by silica gel chromatography using methanol/ CH_2Cl_2 as an eluent to give a greenish blue solid (800 mg, 80%). Mp 167 -169 ^{o}C. ^{1}H NMR (400 MHz, DMSO-d_6) **δ** 1.25 (t, 6H, *J* = 7.0 Hz), 1.63 (s, 12 H), 1.73 – 1.81 (m, 2H), 2.51 – 2.56 (m, 4H), 3.09 – 3.14 (m, 4H), 3.68 – 3.73 (m, 4H), 4.10 (q, 4H, *J* = 7.0 Hz), 5.95 (d, 2H, *J* = 13.4 Hz), 7.12 – 7.18 (m, 2H), 7.23 (d, 2H, *J* = 7.8 Hz), 7.33 – 7.39 (m, 2H), 7.53 (d, 2H, *J* = 7.3 Hz), 7.61 (d, 2H, *J* = 13.4 Hz). ^{13}C NMR (400 MHz, DMSO-d_6): **δ** 12.1, 21.8, 25.0, 28.7, 38.3, 46.9, 48.1, 54.7, 96.2, 110.1, 122.7, 123.6, 124.1, 128.8, 140.6, 140.7, 142.6, 168.2. LC MS (*m/z*): 560.9 (M^{+})

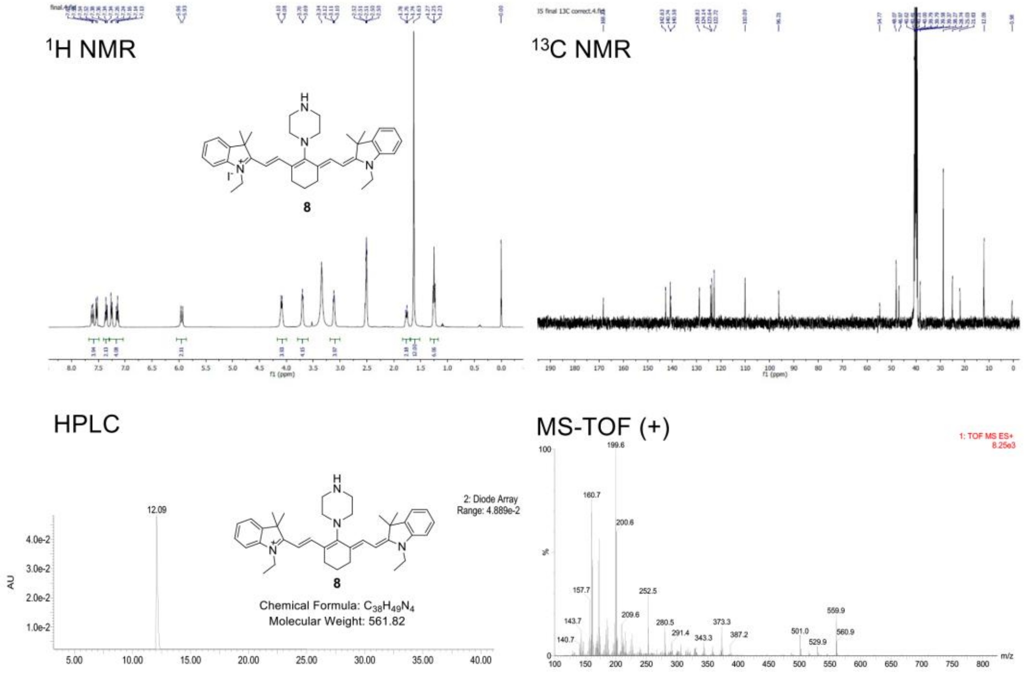

Figure S1. Chemical analyses of **PH08** including ^{1}H and ^{13}C NMR, HPLC, and TOF-MS.

2-((E)-2-((E)-2-(4-((2,4-dinitrophenyl)sulfonyl)piperazin-1-yl)-3-(2-((E)-1-ethyl-3,3-dimethylindolin-2-ylidene)ethylidene)cyclohex-1-en-1-yl)vinyl)-1-ethyl-3,3-dimethyl-1-indol-1-ium, iodide (**9**)**.** To an anhydrous solution of compound **8** (230 mg, 0.40 mmol) in dichloromethane and acetone (1:1), 2,4-dinitrobenzenesulfonyl chloride (1.2 equiv.) was added portion wise at 0°C in the presence of a catalytic amount of triethylamine under a nitrogen atmosphere. After completion of the reaction as confirmed by TLC and UV, the reaction mixture was cooled to room temperature and the solvents were removed under reduced pressure. The crude product was purified by silica gel column chromatography eluted with CH_2Cl_2/CH_3OH (9:1, v/v). The pure product was obtained in an 80% yield. Mp: 219 -222 ^{0}C. δ ^{1}H NMR (400 MHz, DMSO-d_6):1.26 (t, 6H, J = 6.5 Hz), 1.51 (s, 12H), 1.71 – 1.79 (m, 2H), 3.49 – 3.53 (m, 4H), 3.59 – 3.69 (m, 4H), 4.08 – 4.19 (m, 4H), 6.05 (d, 2H, J = 13.5 Hz), 7.17 – 7.23 (m, 2H), 7.31 – 7.41 (m, 4H), 7.50 (d, 2H, J = 13.5 Hz), 8.42 (d, 1H, J = 8.8 Hz), 7.73 – 7.83 (dd, 1H, J = 2.5 Hz, 8.8 Hz), 9.28 (d, 1H, J = 2.5 Hz). ^{13}C NMR (400 MHz, DMSO-d_6): δ 12.4, 25.1, 28.1, 30.9, 48.6, 50.9, 53.4, 87.8, 110.8, 122.8, 125.8, 141.1, 141.9, 142.2, 148.5, 151.2, 169.7. LC MS (*m/z*): 790 (M^{+}-1).

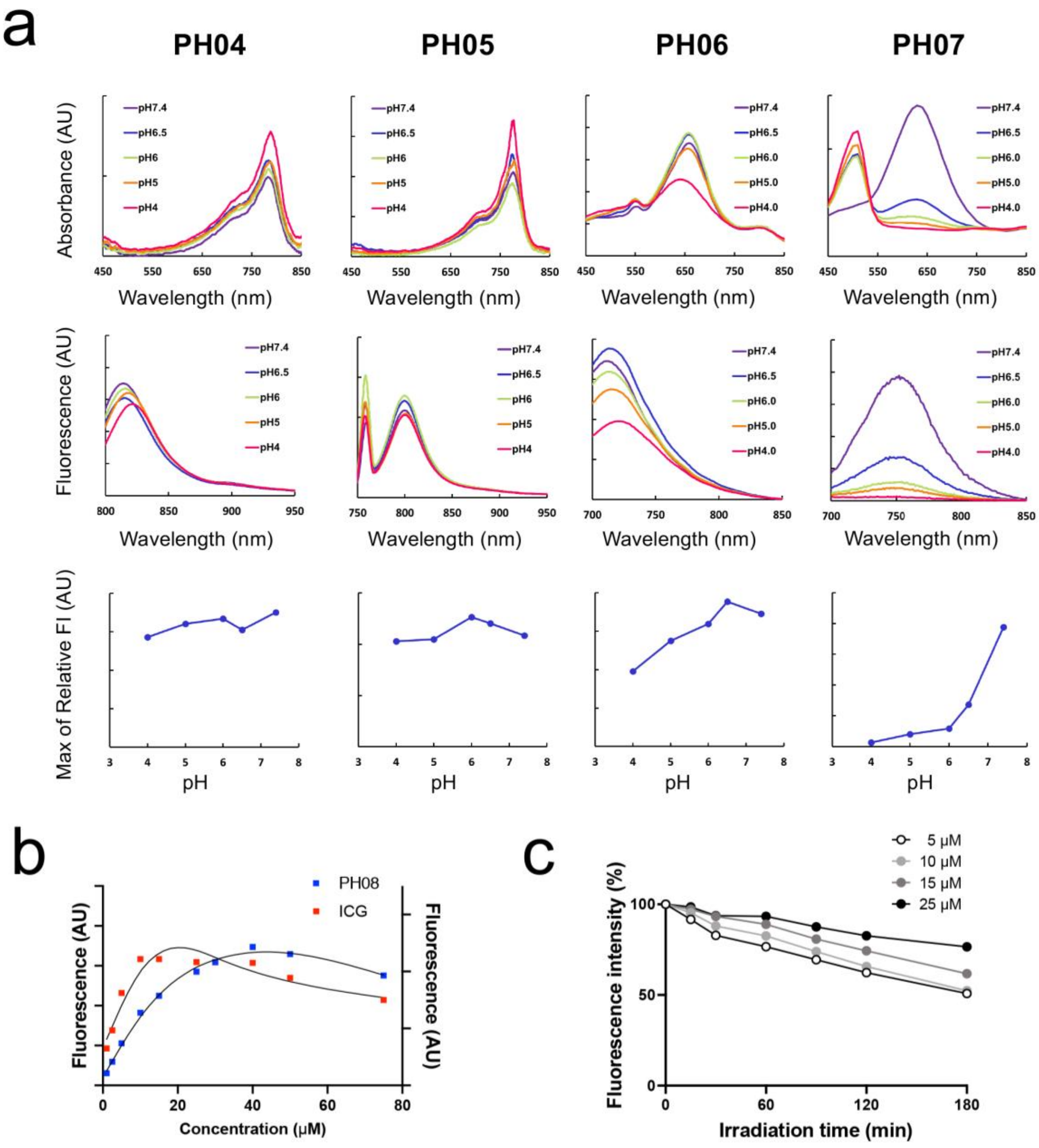

Figure S2. Optical properties of candidate fluorophores. (a) pH-dependent changes in absorption and fluorescence emission were determined in phosphate-buffered saline (PBS) with 5% bovine serum albumin (BSA). (b) Quenching patterns of PH08 and ICG in terms of concentration measured using the FLARE imaging system after incubating the fluorophore in 5 % BSA. (c) Photobleaching curves were obtained by incubating different concentrations of PH08 in 5% BSA for 3 h under continuous laser irradiation.

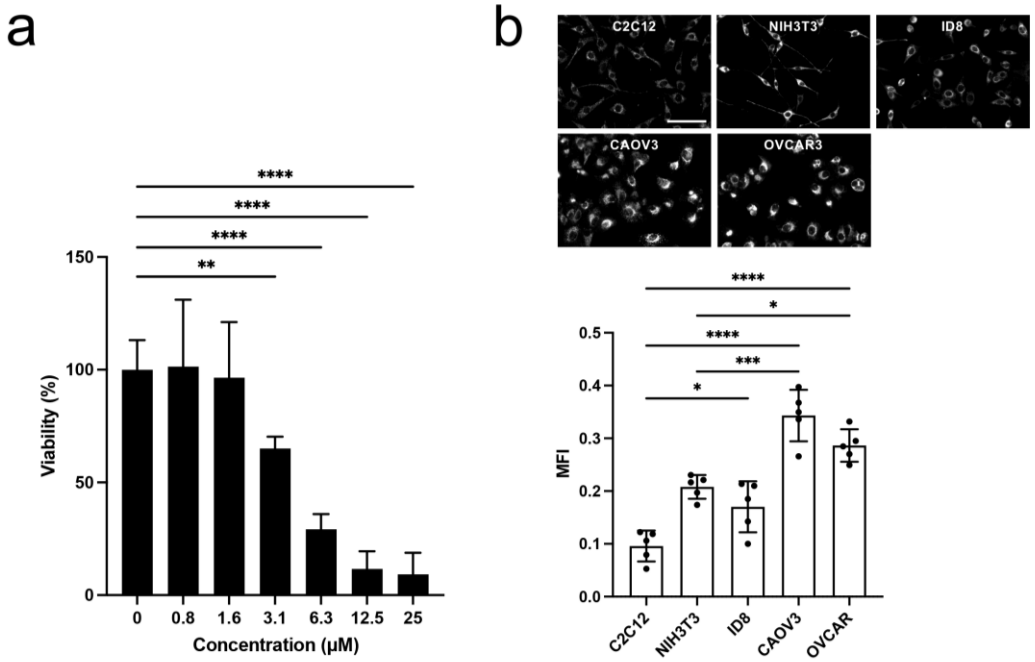

Figure S3. Tumor cell targetability and cytotoxicity of the pH sensing NIR fluorophore. (a) Cytotoxicity of the fluorophore. Cells were treated with 0-25 μM of PH08 for 24 h, followed by an assessment of cell viability using the Cell Counting Kit-8 (CCK-8) (n = 3, mean ± s.d.). (b) Murine and human ovarian cancer cell lines including ID8 cells and SKOV3 cells, NIH/3T3 fibroblast, and C2C12 muscle cells were cultured and incubated at 37°C for 15 min in the presence of 0.2 μM PH08 and imaged under the epifluorescence NIR microscope. Quantitative measurements of the fluorescence intensity of cells in 10% FBS (n = 3, mean ± s.d.). Scale bar = 100 μm. ns, not significant, *$P < 0.05$, **$P < 0.01$, ***$P < 0.001$, ****$P < 0.0001$ by one-way ANOVA followed by Tukey's multiple comparisons test.

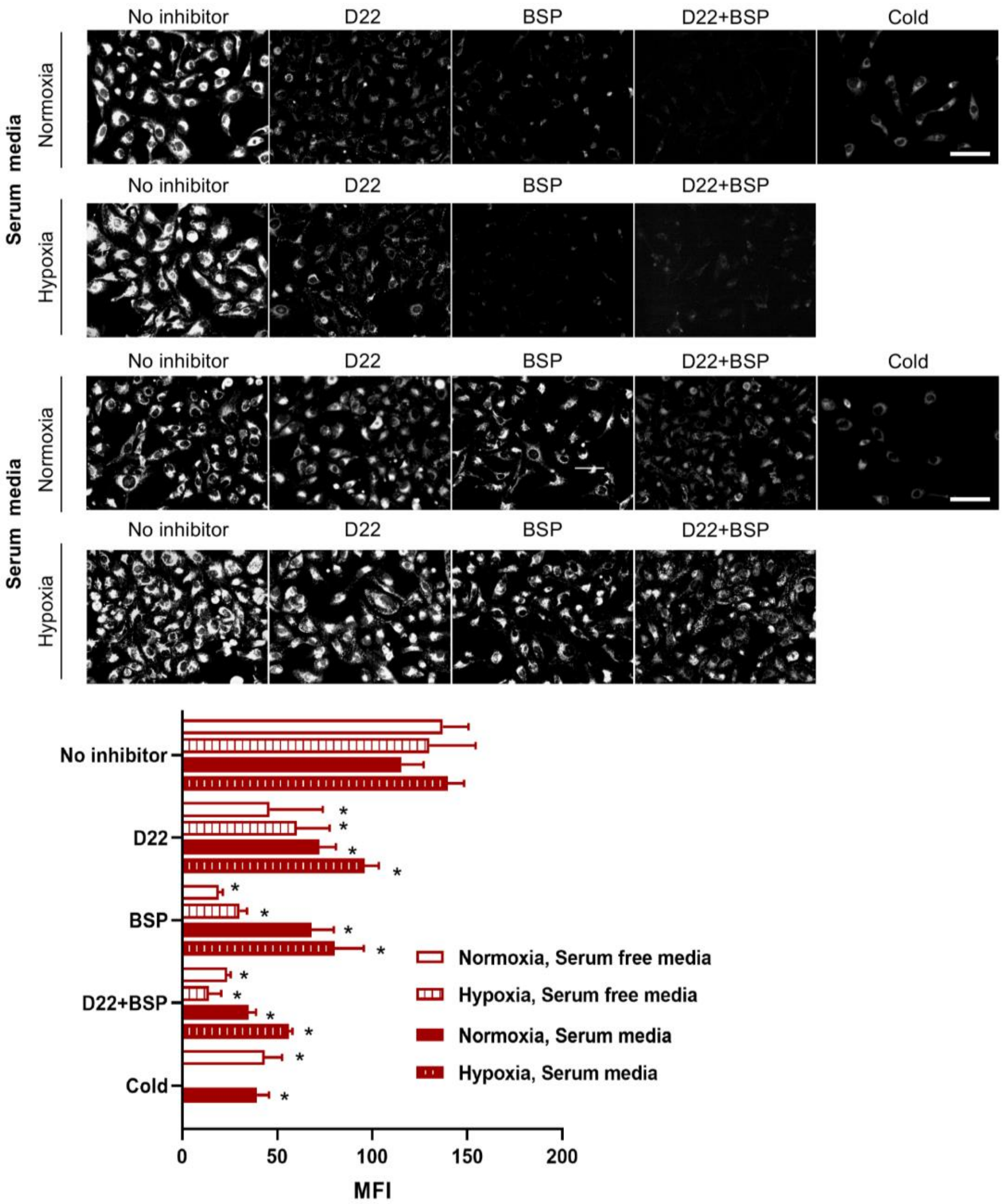

Figure S4. Cellular uptake of pH sensing NIR fluorophores via membrane transporters in a human ovarian cancer cell line. *In vitro* tumor cell uptake of the pH sensing NIR probes with or without serum in media under normoxic and hypoxic conditions. An inhibition assay of cellular uptake of PH08 was performed to determine the entry mechanisms. SKOV-3 human cancer cells were incubated under normoxic and hypoxic (1% O_2) conditions. Cultured cells were incubated with BSP, D22, or both inhibitors for 10 min and then incubated with 0.1 µM PH08 in 10% or 0% FBS for 15 min. Alternatively, cells were incubated at 4 °C for 30 min. Cells were then imaged under the epifluorescence NIR microscope. (**Top**) Representative fluorescence images of cells are shown. The contrast was normalized across all images. Scale bar = 100 µm. (**Bottom**) Quantitative measurements of the fluorescence intensity in cells (n = 3, mean ± s.d.). $^*P < 0.05$ by one-way ANOVA followed by Tukey's multiple comparisons test.

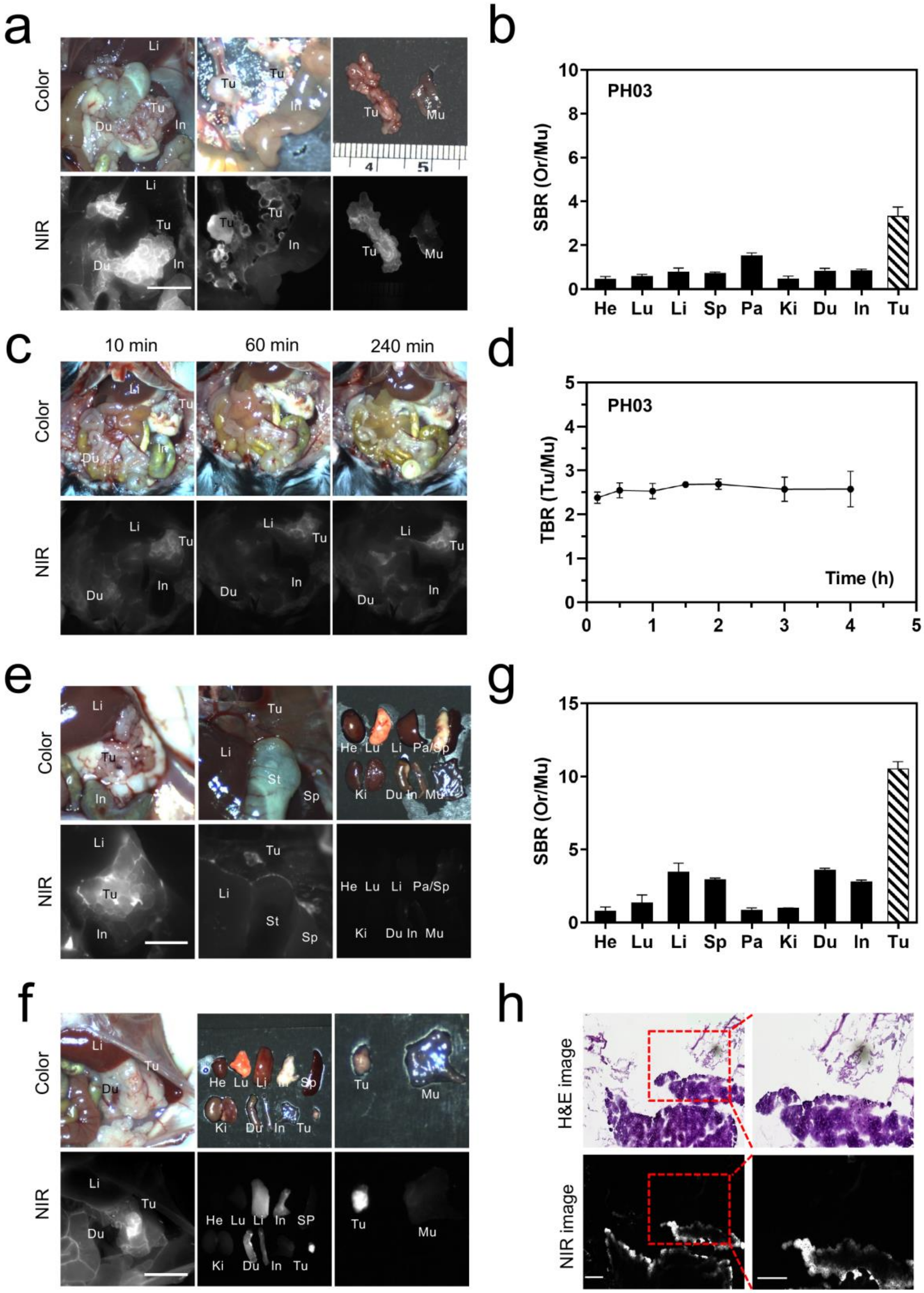

Figure S5. ***In vivo*** **evaluation of tumor cell targetability of pH sensing NIR fluorophores in a mouse model of ovarian cancer**. (a) Intraoperative color and NIR fluorescence images of the abdominal cavity and peritoneal dissemination of ovarian cancer and major organs 4 h post-

intraperitoneal injection of 20 nmol of PH03. Scale bar = 5 mm. (b) Quantitative analysis of target-to-background ratio (TBR) of peritoneal tumors and major organs for PH03. TBR was determined by comparing the signals of tumors (Tu) or organs against muscle (Mu). Du, duodenum; He, heart; In, intestine; Ki, kidneys; Li, liver; Lu, lungs; Pa, pancreas; Sp, spleen; St, stomach (n = 3, mean ± s.e.m.). (c,d) Quantitative time-course assessment of TBR for up to 240 min post-injection of PH03 (n = 3, mean ± s.e.m.). (e) *In vivo* and *ex vivo* biodistribution of PH08 in major organs. 20 nmol of PH08 was injected intraperitoneally 4 h prior to imaging and resection. Abbreviations used are: Du, duodenum; He, heart; In, intestine; Ki, kidneys; Li, liver; Lu, lungs; Mu, muscle; Pa, pancreas; Sp, spleen; St, stomach; Tu, tumors. Scale bars = 5 mm. (f) Intraoperative color and NIR fluorescence images of the abdominal cavity and peritoneal dissemination of human ovarian cancer SKOV3 4 h post-intraperitoneal injection of 20 nmol of PH08 in a xenograft model. Scale bar = 5 mm. (g) Quantitative analysis of TBR of peritoneal SKOV3 tumors and major organs for PH08 (n = 3, mean ± s.e.m.). (h) Histological analysis of tumor-targeted PH08 in the orthotopic ovarian cancer model. Representative hematoxylin and eosin (H&E) and corresponding NIR fluorescence images are shown. Scale bars = 50 µm.

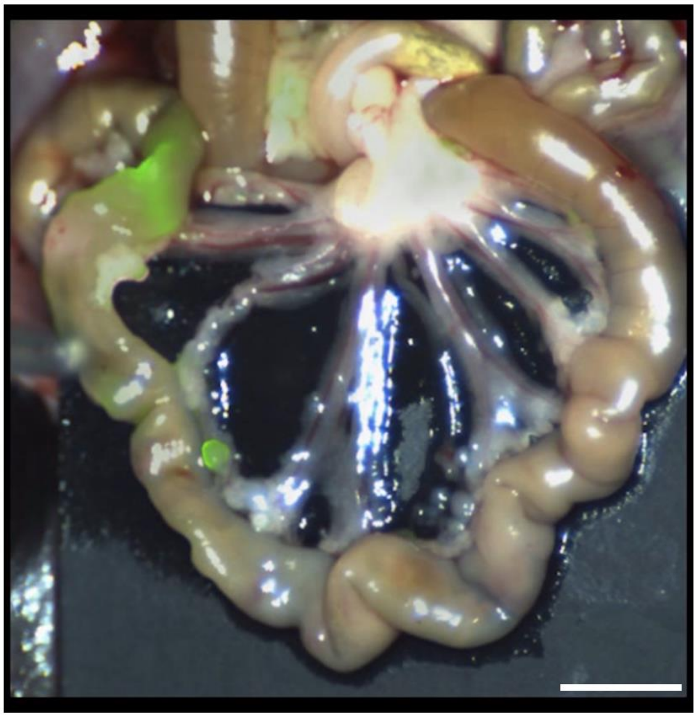

Supplementary Movie S1. Fluorescence-guided surgery of ovarian cancer using PH08. 20 nmol of PH08 was injected intraperitoneally and intraoperative imaging and FGS was performed 4 h post-injection. Scale bar = 5 mm. 3x speed.

REFERENCES

[1] A. Levitz, F. Marmarchi, M. Henary, *Molecules* **2018**, *23*.

[2] A. Levitz, F. Marmarchi, M. Henary, *Photochem Photobiol Sci* **2018**, *17*, 1409.

[3] X. Ma, M. Laramie, M. Henary, *Bioorg Med Chem Lett* **2018**, *28*, 509.
